# Placebo controlled drug provocation testing (PCDPT) to betalactam and non-betalactam antibiotics and its diagnostic value in drug allergy diagnostics

**DOI:** 10.1186/2045-7022-4-S3-P58

**Published:** 2014-07-18

**Authors:** Galina Balakirski, Stefani Roeseler, Gerda Wurpts, Hans F Merk

**Affiliations:** 1 University Hospital of Aachen, Department of Dermatology & Allergology, Germany

## Background

In contemporary drug allergy diagnostics PCDPT holds significant value. We analyzed whether results of PCDPT correlate with the results of other diagnostic procedures such as *in vivo* skin testing and *in vitro* laboratory tests on betalactam and non-betalactam antibiotics performed in our department during the last 5 years.

## Method

All the patients underwent PCDPTs to betalactam and non-betalactam antibiotics from the 1st of October 2008 to the 1st of October 2013 in our department reported about one or more adverse side reactions to antibiotic in the background and underwent standard diagnostic procedures prior PCDPT. We compared the results of diagnostic procedures such as *in vivo* skin testing and *in vitro* laboratory tests to the results of PCDPT.

## Results

PCDPTs to antibiotics were performed either to confirm (11% of cases) or to deny (55% of cases) the diagnosis of antibiotic allergy or to find an alternative drug if the allergy to certain antibiotics was confirmed by prior diagnostics (34% of cases). PCDPTs to antibiotics were performed in 56 patients: 18% were to classical betalactam antibiotics (without cephalosporins)and 82% to non-betalactam antibiotics. In 89% of cases no reaction to both placebo and drug was seen. 7% of PCDPTs showed objectified reaction to drug, 4% a non-objectifiable or questionable reaction to drug and no cases of a non-objectifiable reaction to placebo without any reaction to drug were seen. Prior to PCDPT we performed available *in vivo* skin testing and *in vitro* laboratory tests. In 42% of cases we measured specific IgE antibodies to antibiotics and in all cases they were negative. In 32% of patients we performed a basophil activation test and in all cases it was negative. In 90% of cases we performed a skin prick test, 94% of tests were negative, in 4% dermatographic urticaria was seen and in 2% reaction was questionable. We performed intracutaneous test in 56% of cases, in 98% it was negative and in 2% a questionable reaction was observed. In 63% of patients a patch test was performed and in all cases it was negative. In all cases that patients showed a positive objectified reaction to the antibiotic in PCDPT the tests performed prior to PCDPT were negative.

## Conclusion

PCDPT remains a gold standard in drug allergy diagnostics. *In vivo* skin testing and *in vitro* laboratory tests may be falsely wrong and therefore it requires additional PCDPT for a final diagnostic statement.

